# Mapping sheep to human brain: The need for a sheep brain atlas

**DOI:** 10.3389/fvets.2022.961413

**Published:** 2022-07-29

**Authors:** Ashik Banstola, John N. J. Reynolds

**Affiliations:** ^1^Department of Anatomy, School of Biomedical Sciences, University of Otago, Dunedin, New Zealand; ^2^Brain Health Research Centre, University of Otago, Dunedin, New Zealand

**Keywords:** sheep, brain, atlas, template, translational neuroscience, stereotaxic coordinates

## Abstract

A brain atlas is essential for understanding the anatomical relationship between neuroanatomical structures. Standard stereotaxic coordinates and reference systems have been developed for humans, non-human primates and small laboratory animals to contribute to translational neuroscience research. Despite similar neuroanatomical and neurofunctional features between the sheep and human brain, little is known of the sheep brain stereotaxy, and a detailed sheep atlas is scarce. Here, we briefly discuss the value of using sheep in neurological research and the paucity of literature concerning the coordinates system during neurosurgical approaches. Recent advancements such as computerized tomography, positron emission tomography, magnetic resonance imaging, functional magnetic resonance imaging and diffusion tensor imaging are used for targeting and localizing the coordinates and brain areas in humans. Still, their application in sheep is rare due to the lack of a 3D stereotaxic sheep atlas by which to map sheep brain structures to its human counterparts. More recently, a T1- and T2-weighted high-resolution MRI 3D stereotaxic atlas of the sheep brain has been generated, however, the journey to create a sheep brain atlas by which to map directly to the human brain is still uncharted. Therefore, developing a detailed sheep brain atlas is valuable for the future to facilitate the use of sheep as a large animal experimental non-primate model for translational neurological research.

## Introduction

A brain atlas is a valuable tool containing pictures of brain sections in different anatomical orientations (three-dimensional space; coronal, sagittal and axial planes), with the coordinates of relevant brain structures to define their outlines or volumes. An atlas enables the researcher to calculate stereotaxic coordinates for a variety of stereotaxic procedures to accurately target deep brain structures for recording or lesioning (1–4). This atlas when combined with electrophysiological data helps to better understand the functional activity in brain networks across species (5). Having a highly precise and consistent atlas assures consistency in defining the boundaries of various brain structures between publications from different researchers (6). In addition, atlas and templates are necessary to improve understanding of neuroanatomical structures, leading to advancements in the field of neurology, and neurosurgery and to aid translational neuroscience research.

**Figure 1:**
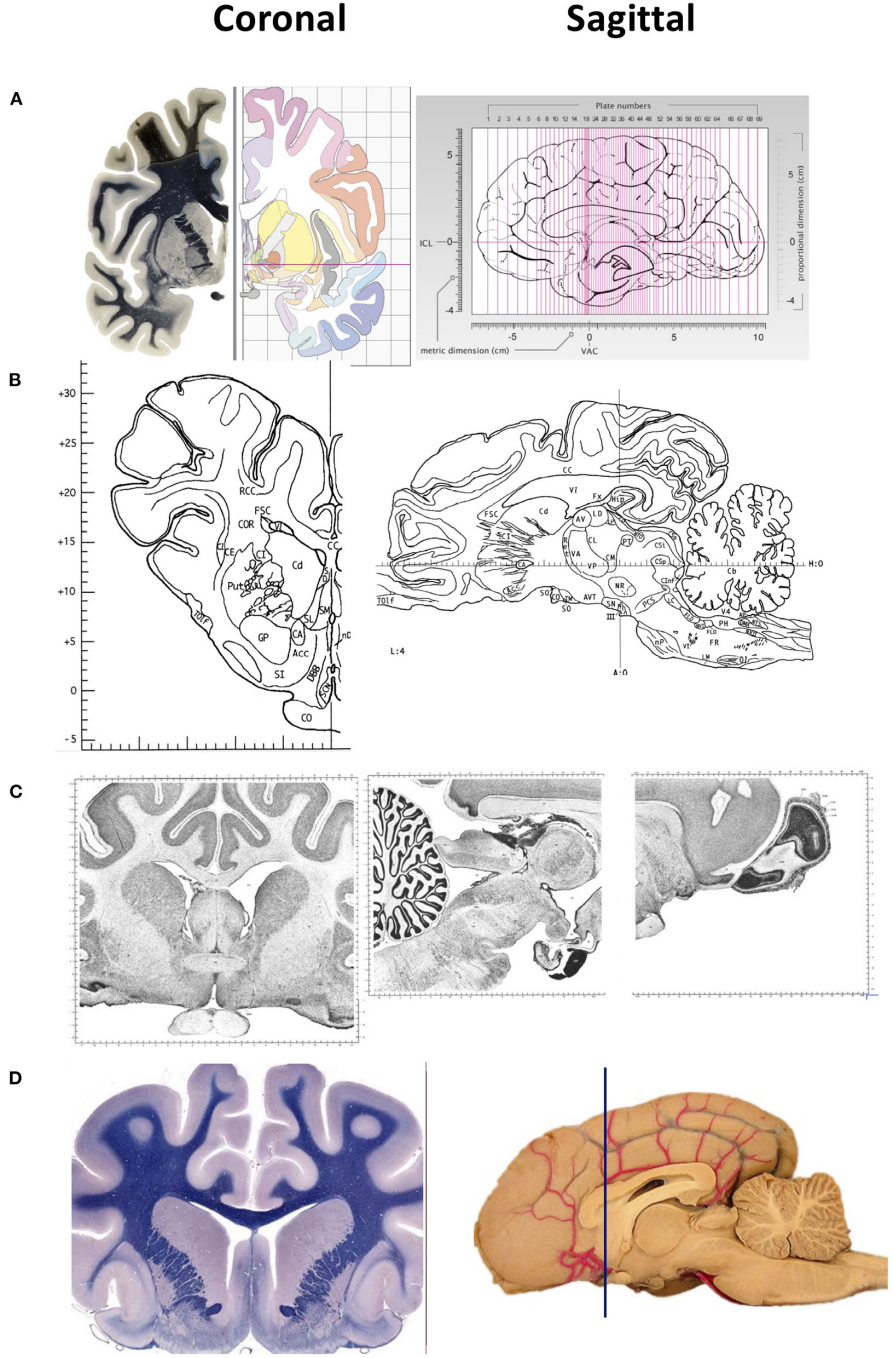
Representative images of two-dimensional sections of the brain, coronal (right) and sagittal (left) sectons of brains of various species. **(A)** Human brain. Coronal sections show the fiber tracts in the left panel and schematic diagrams in the right panel. The sagittal section shows the plane of the 69 sections depicted in the atlas. The intercommisural line (ICL) and the vertical line (VCA) pass through the center of the anterior and posterior commissure, and the center of the anterior commissure, respectively. **(B)** Pig brain. Coronal and sagittal sections in this example show the coordinates 14.50 mm ahead of the posterior commissure and 4.00 mm laterally from the midsagittal plane, respectively. **(C)** Cat brain. Coronal and sagittal sections are examples of Nissl stained sections from the adult cat (Felis Catus). **(D)** Dog brain. Coronal and sagittal sections are shown in the maps of the whole brain from a 5 month old dog (Canis Lupus). The blue line shows the levels at the frontal lobe Images in **(A)** are reproduced from the human brain website https://www.thehumanbrain.info/brain/sections.php. Retrieved May 12, 2022. Images in **(B)** are reproduced from the Stereotaxic atlas of the pig brain by Felix et al. (1) with permission from Elsevier. Images **(C,D)** are reproduced from the brain maps website http://brainmaps.org/ajax-viewer.phpdatid=32&sname=p099-100, retrieved on May 13, 2022.

Among the various animal species used in neuroscience, rodents are the most common. Other used species include domestic animals such as pigs, goats, and sheep, and those that are also companion animals such as horses, dogs and cats. Stereotaxic and automatic tissue segmentation systems with varied detail have been developed for humans, non-human primates, dogs, cats and rodents, but not for sheep. A detailed brain atlas exists for humans, the Allen Human Brain Atlas; for rats, the Rat Brain in Stereotaxic Coordinates (7); for mouse, Allen Brain Atlas: Mouse Brain; for rhesus macaques, NIH Blueprint Non-Human Primate (NHP) Atlas; for long-tailed macaque monkeys (4); for domestic species such as the pig (1), dog (8–10), cat (5), and horse. Some example two-dimensional images of brain coronal and sagittal sections of various animal species is shown in Figure 1. Unfortunately, to our knowledge, no such detailed atlas is available for sheep.

The sheep (*Ovis aries*) is an attractive and convenient animal model for mapping human disorders, particularly for neurosurgical and neuropathological research. Physiological and neuroanatomical similarities between sheep and humans, such as cerebral white matter distribution (11), gyrencephalic cerebral cortexes, thick meninges, and highly distinct sulci and gyri (12–14), make sheep an acceptable large brain animal model for neurological research. Sheep cerebral cortices contain four lobes defined by external landmarks, similar to those of humans (15). Furthermore, the sub-cortical structures in particular, the dorsal striatum, are in two separate sections; caudate nucleus and putamen in sheep, similar to humans (14, 16). In addition, the relatively round skull of the sheep is comparable to the human head, unlike pigs which have a flat and thick skull (17). Therefore, the brains of sheep may have distinct anatomical advantages over small brains for translational research. A detailed review of neuroanatomy is beyond the scope of this paper, but clinically relevant areas are compared briefly in Table 1. Other benefits of using sheep such as greater acceptability to animal ethics committee compared to companion animals and primates, easily available, less expensive, reasonably outbreed, easy management, environmental enrichment not required as they live in their natural pasture, make sheep advantageous an experimental model for translational research over large animal species, in particular primates.

**Table 1:** Comparisons of the central nervous system between sheep and humans.

**Features/Gross**	**Sheep**	**Human**	**References**
Brain shape	Smaller and elongated	Larger and rounded	(18)
Skull thickness (mm)	5.0–6.0	6.5–7.5	(19)
Brain mass (g)	130–140	1,300–1,400	(14)
Four lobes defined by external landmarks	Present	Present	(12)
Sulci and gyri	Present	Present	(12–14)
Cerebral cortex	Primarily neocortex	Primarily neocortex	(13, 20)
Motor cortex	Located in frontal lobe (superior frontal gyrus)	Located in frontal lobe	(21)
Somatosensory cortex	Located in frontal lobe (middle frontal gyrus)	Located in parietal lobe	(21)
Cortical layers	Distinct cellular layers I-VI	Distinct cellular layers I-VI	(22)
Cortical interneuron	Significant role	Significant role	(23)
Frontal lobe	Small	Very large	(18)
Olfactory bulb	Large and well-developed	Small	(24, 25)
Optic chiasm	More pronounced	Less pronounced	
Orbit indentation	Side	Front	(26)
Visual cortex	More lateral	More midline	(26)
White matter	Abundant	Very abundant	(27)
Cerebrum	More elongated	Less elongated	(18)
Rigid tentorium cerebelli	Present	Present	(28)
Cerebellum	Smaller, located posteriorly (behind the cerebrum)	Larger, located caudally	(29)
Meninges	Thick, well-developed	Thick, well-developed	(18)
Subventricular zone	Laminar structure	Laminar structure	(30)
Subgranular Zone	Laminar structure	Laminar structure	(30)
Hippocampus	Ventral aspects of cerebrum	Ventral aspects of cerebrum	(24)
Basal ganglia	Separate caudate and putamen	Separate caudate and putamen	(14, 16)
Substantia nigra pars compacta and pars reticulata cell diameter (μm)	9–26 and 10–23	14–50 and 20–30	(22, 31)
Substantia nigra pars compacta and pars reticulata average volume (mm^3^)	13 and 152	68	(22, 31)
Substantia nigra pars compacta and pars reticulata average cell number	39,481 and 51,800	436,000	(22, 31)
Gross spinal cord	Located posteriorly	Located caudally	(18)
Vertebral bodies (Cervical spine)	Taller than wide	Wider than tall	(32)
Lumbar spine curvature	Kyphotic	Lordotic	(32)
Spinal canal width	Wider (Identical to human)	Wider	(32)
Spinous process (cervical and thoracic regions)	Longer	Smaller	(32)
Spinous process (Lumbar regions)	Longer	Longer	(32)
Sciatic nerve origin	L6-S2	L4-S3	(33)
Pineal gland	Large and round; located at the interface between the cerebral hemispheres and cerebellum, not lobulated	Small, pine cone shaped, located within the posterior wall of the third ventricle near the center of the brain, lobulated	(34)

*Where no journal reference is given, the human features/gross are adopted for comparison with sheep data from the Nervous System by Snyder et al. (18) with permission from Elsevier*.

Despite similar neuroanatomical and neurofunctional features between sheep and human brains, and the relatively low cost of the sheep as a model for teaching human-like brain anatomy, very little is known about the ovine brain anatomical relationships, and a detailed sheep atlas is scarce. The only available atlases are either limited to specific brain areas (35), consist of a series of gross photographs of sheep brain (36), or provide labeled coronal sections (both cell and fiber stain) but do not offer standard stereotaxic coordinates and reference system (e.g., The Michigan histological sheep brain atlas; https://brains.anatomy.msu.edu/brains/sheep/index.html; retrieved on May 12, 2022). Although there is a sheep atlas with a coordinate system (covering a few brain areas), histology sections with labeled brain structures (as in the Michigan histological data atlas; but not the standard stereotaxic coordinates/reference system) and neuroimaging atlas there is no comprehensive atlas that actually combines all these features to enable defined regions to be easily localized. Therefore, the neuroscience research community would be benefitted by having a detailed sheep atlas that combines both neuroanatomical regions on both histology (as in the Michigan histological data atlas) as well as neuroimaging (MRI atlases)to enable defined regions to be easily localized.

## Brief history of resources developed for the study of the sheep brain

The study of the cortico-spinal or pyramid tract fibers of the sheep brain can be dated back to the late 18th century (the literature refers to 1877 by Atruro Maracacci)(37). Researchers made strenuous efforts to map the brain and anatomical areas and to partition these based on cytoarchitectonic information available at that early stage. Most of the early studies in sheep were based on electrical or mechanical stimulation of the different brain regions and mapping with a pattern of body response to stimuli (37–39). In 1967, Richard published the first hard copy sheep atlas, “*Atlas stereotaxique du cerveau de beri*a.” However, the atlas was written in the French language and was limited to specific brain areas, including subcortical structures such as the thalamus, hippocampus, and hypothalamus (35). The stereotaxic brain atlas in English was prepared by McKenzie and Smith in 1973 from female merino sheep and was used by others in guiding electrical stimulation of the brain, diencephalic lesions, and intracerebroventricular chemical stimulations (40). Opdam and colleagues, first reported the sheep (merino) model of focal epilepsy (penicillin-induced) in 2002 using concurrent EEG and fMRI (41). The localization of coordinates for surgical placement and electrodes was based on the anatomic atlas of the sheep brain, however, the authors did not relate the details of the stereotaxic reference systems to the distinct anatomical structures identified from their experimental findings to help map the findings to the structures found in the human brain.

### Recent advancement

Recent advances in mapping technology have made it possible to study neuroanatomical features and localization of brain areas in a variety of animals, including sheep (3, 42–44). The established techniques for targeting brain areas and localizing the coordinates such as CT scan, PET, MRI, fMRI, and DTI are widely used in humans. The neuroimaging techniques are being applied to sheep, albeit not extensively because they fit into conventional scanners and MRI units due to their comparable body size, skulls and brain volumes to humans (14, 28, 43, 45).

In 2014, frameless MRI guided stereotactic access to the ovine brainstem was developed and validated using the modified Brainsight^TM^ stereotactic system (46). Nevertheless, the approach was limited to the midbrain and pons on post-mortem imaging on sheep heads. Nitzsche et al. (44) published an MRI-based ovine brain template with tissue probability maps offering a detailed stereotaxic reference frame to localize brain areas and anatomical features. However, this ovine brain atlas was limited to cerebral morphology and tissue volume, mainly gray matter, white matter, cerebrospinal fluid (CSF), cerebral peduncle, and pons. Other critical anatomical structures such as the cerebellum, medulla oblongata, olfactory bulb and subcortical brain areas were not included.

Russell et al. (47) used CT scanning and 3D reconstruction techniques to study intracranial volume loss as well as regional neurodegeneration over time (between 5 and 13 months and 11 and 15 months for CLN5 and CLN6 sheep Baten disease models, respectively) in occipital lobes and propagation throughout the cerebral cortex. The CT images included olfactory bulbs in the cerebellum. Nevertheless, details of inner and external sheep brain structures was again absent from these works.

3D high-resolution MRI sheep brain templates have been constructed for multi-institution neuroimaging studies using living animals. More recently, Ella and colleagues generated the first complete T1 and T2-weighted high-resolution MRI 3D stereotaxic atlas of an *in vivo* sheep brain (2, 48). This elegant 3D atlas defined an MRI stereotaxic coordinate system, probability maps and templates of CSF, gray and white matter. In addition, the 3D atlas also demonstrated 25 cortical and 28 subcortical brain structures. However, the journey of mapping the coordinates of sheep brain structures identified in atlases such as this to that of the human brain still needs to advance, thereby providing a missing tool for translational research.

Clarke and Whitteridge, (49) made their own atlases for the cortex to study the cortical visual areas (Visual I and II) of the sheep for the investigation of controlled eye movement. In their work, they considered the coordinates used were relative to the interaural plane in the usual way but the details of the coordinates for targeting the visual cortex were missing. However, they have referred to the Richard atlas for the mid-brain. The visual cortex was found to be thin in sheep (1.488 mm) compared to those of macaques (1.657 mm), and chimpanzees (2.109 mm) (50).

Bombardi et al. (51) studied the organization of the lateral nucleus of sheep amygdaloid complex which is little known in ruminants. Due to the lack of a proper sheep brain atlas, the delineation of the nuclear boundaries of the amygdala was based on the image sections found in the University of Wisconsin and Michigan State Comparative Mammalian brain collections.

More recently, DTI, mathematical and image-analysis techniques, of the intracranial pyramidal and extrapyramidal tracts, were applied to study the laminar organization and projections of the sheep motor cortex. The results showed the comparable thickness of the cortex and other morphometric values to other mammalian species including the chimpanzee. Somatotopic mapping however was taken from early studies by Simpson and King due to the lack of a currently detailed sheep atlas (52). Mapping orbitofrontal cortex (OFC) and its connection to brain areas of the sheep with the chimpanzee and humans was investigated by Tommaso et al. using DTI. The authors found a higher number of cortico-cortical fibers connecting the visual areas with OFC similar to that of the human brain (53).

## Conclusion

At least 80–90% of novel therapeutic agents tested on many rodents are found to be ineffective when translating therapies into clinical research. One of the factors could be the inappropriate selection of a suitable experimental animal model for translational research. In addition, regulatory authorities expect therapeutic agents and medical device testing on at least one small rodent and large non-rodent gyrencephalic species prior to clinical trials. As neuroscience becomes increasingly advanced, highly accurate and consistent brain atlases are needed for high-precision neuroscience experimentation. The neuroscience research community would be benefitted by having a detailed ovine brain atlas with coordinates that combine both neuroanatomical regions on both histology (as in the Michigan histological data atlas) as well as neuroimaging (MRI atlases) to enable defined regions to be easily localized. Although there are some still existing limits such as the scarcity of neurochemical and electrophysiological data, as well as the difficulty and high cost of performing transgenesis experiments, we believe that creating a detailed ovine brain atlas consisting of well-defined brain regions (easily identifiable) and somatotopic organization (similar to the available human and rodents atlas) is necessary for clinically relevant translational neuroscience and neurological research. Such an atlas would allow for detailed regional analysis and easier surgical manipulation, facilitate the generalizability and comparability of experimental results across studies and laboratories and reduce the requirement for higher resolution imaging. This would be a significant piece of research. Such a tool would be extremely valuable for research in the next 5 to 10 years using sheep as a large animal non-primate model for translational neurological research.

## Author contributions

Conceptualization and writing—original draft: AB. Visualization and writing—review and editing: AB and JR. All authors contributed to the article and approved the submitted version.

## Funding

This work was supported by the Neurological Foundation New Zealand grant 2130PRG.

## Conflict of interest

The authors declare that the research was conducted in the absence of any commercial or financial relationships that could be construed as a potential conflict of interest.

## Publisher's note

All claims expressed in this article are solely those of the authors and do not necessarily represent those of their affiliated organizations, or those of the publisher, the editors and the reviewers. Any product that may be evaluated in this article, or claim that may be made by its manufacturer, is not guaranteed or endorsed by the publisher.
